# Association of the VDAC3 gene polymorphism with sperm count in Han-Chinese population with idiopathic male infertility

**DOI:** 10.18632/oncotarget.16891

**Published:** 2017-04-06

**Authors:** Lianjun Pan, Qingzhen Liu, Jingyun Li, Wei Wu, Xinru Wang, Dan Zhao, Jiehua Ma

**Affiliations:** ^1^ State Key Laboratory of Reproductive Medicine, Department of Urology, Nanjing Maternity and Child Health Care Hospital Affiliated to Nanjing Medical University, Nanjing 210004, China; ^2^ Department of Anesthesiology, Jinling Hospital, School of Medicine, Nanjing University, Nanjing 210002, China; ^3^ Department of Gynaecology, Nanjing Maternity and Child Health Care Hospital Affiliated to Nanjing Medical University, Nanjing 210004, China; ^4^ State Key Laboratory of Reproductive Medicine, Institute of Toxicology, Nanjing Medical University, Nanjing 210029, China

**Keywords:** VDAC, male infertility, semen

## Abstract

Voltage-dependent anion channel (VDAC) is a multifunctional channel protein across the outer mitochondrial membrane of somatic cells and participates in many physiological and pathophysiological processes. Up to now, only a few studies, including our previous studies, showed that VDAC exists in mammalian spermatozoa and is involved in spermatogenesis and sperm functions. There is no report about VDAC genetic variants in germinal tissues or cells. To investigate the possible association between VDAC genetic variants and human sperm quality, we performed semen analysis and variant Genotyping of VDAC3 subtype (rs7004637, rs16891278 and rs6773) of 523 Han-Chinese males with idiopathic infertility respectively by computer assisted semen analysis (CASA) and single nucleotide polymorphism (SNP) Genotyping assay. No significant association was found between rs7004637 and rs6773 genotypes and semen quality. However, the AG genotype of rs16891278 showed a significantly lower sperm concentration compared with the AA genotype (*P* = 0.044). Our findings suggest that VDAC3 genetic variants may be associated with human sperm count.

## INTRODUCTION

Voltage-dependent anion channels (VDAC), also known as mitochondrial porins, is first identified in the outer mitochondrial membrane of Paramecium aurelia [1]. Then it is found to be mainly located on the mitochondrial membrane of somatic cells of all eukaryotes [2, 3]. The mammalian VDAC gene family consists of three homologous genes (VDAC1, 2, 3), each of them shares approximately 70% identity to each other [4] and expresses three different proteins [5, 6]. VDAC participates in many physiological and pathophysiological processes (energy metabolism, cell apoptosis, and so on) through regulating membrane permeability to small ions and molecules (e.g. Na+, Ca2+, Cl–, ATP, glutamate) [7–12]. However, the exact function of each VDAC subtype remains controversial.

Although VDAC has been extensively studied, only a few studies focus on the localization and functions of VDAC in male germinal tissues and cells. A few studies now available about VDAC in mammalian reproductive tissues and cells have suggested that VDAC might play important roles in spermatogenesis, motility, capacitation and acrosome reaction [13, 14]. The current studies suggest that VDAC1 is mainly localized in Sertoli cells, and VDAC2 and VDAC3 are present in germ cells [6, 13, 14]. Abundant VDAC3 is also found in Leydig cells [6]. In mature spermatozoa, VDAC2 and VDAC3 are also observed to be localized abundantly in bovine outer dense fiber (ODF) of the flagella [15]. VDAC2 is also found in plasma membrane or acrosomal membrane of sperm head [16]. Functionally, VDAC2 and VDAC3 are involved in spermatogenesis and sperm functions [16, 17]. Recent series of studies demonstrated that VDAC exists in human spermatozoa and may be participate in acrosome reaction [18, 19].

In three isoforms, VDAC3 is the most widely distributed in germinal tissues and cells, and different in pore-forming ability comparing with others. Furthermore, male mice lacking VDAC3 show markedly reduced sperm motility and are infertile despite having normal testes and sperm numbers [14]. Taken together, these previous observations suggest that VDAC3 may be more important for male fertility, raising an important question of whether variants in this gene are associated with diminished semen quality.

VDAC3 is located on chromosome 8p11.2, spanning 13.3 kbp and is made up of 9 exons [22]. It is commonly known that a variety of mutations in a gene can influence gene expression and in turn affect the biological impact [23]. Single nucleotide polymorphisms (SNPs) represent the most abundant class of inheritable human gene mutations, and have profound influence on gene expression and function [24, 25]. VDAC3 gene has been extensively studied in somatic cells, but there is only scant information about the association between VDAC3 and male fertility. Moreover, up to now, there is no related report on the association between VDAC3 genetic variants and semen quality. Here, we investigated the frequency distribution of three tagSNPs representing genetic variation across the entire VDAC3 gene in 523 patients with idiopathic infertility. This study was focused on semen quality, which is the predominant manifestation of male infertility [26]. Therefore, the objective of this study is to investigate the possible association between the genetic variants of VDAC3 and semen quality in 523 males with definite idiopathic infertility.

## RESULTS

This study contained 523 ethnic Han-Chinese males with definite idiopathic infertility at 31.75 ± 5.47 years of age. The mean duration of sexual abstinence before semen collection was 4.88 ± 2.66 days. The sperm parameters between the stratification of selected characteristics are listed in Table 1. Briefly, no significant differences were found in the stratification of age for all the six parameters. However, the sperm concentration was significantly higher in the ever smoking group when compared with the never smoking group. The semen volume was significantly lower in the ever drinking group when compared with the never drinking group. For BMI, the ≥ 25 group had significantly lower VCL, VSL and VAP compared with the < 20 group. For the duration of sexual abstinence, the 4–7 days group and ≥ 7 days group were associated with a significantly higher semen volume. Additionally, as for the ≥ 7 days group, the significantly higher concentration and lower motility was found when compared with the < 4 days group.

**Table 1:** Association between the selected individual characteristics and sperm parameters in 523 males with definite idiopathic infertility

	*N* (%)	Semen volume (mL)	Concentration^a^	Motility (%)	VCL (μm/s)	VSL (μm/s)	VAP (μm/s)
**Age**							
< 29	145 (27.72)	3.23 ± 1.28	3.80 ± 1.15	46.39 ± 26.26	34.04 ± 13.18	20.29 ± 7.90	22.69 ± 8.66
29-33	181 (34.61)	3.29 ± 1.24	3.76 ± 1.11	47.38 ± 24.56	35.54 ± 14.58	21.23 ± 8.93	23.53 ± 9.61
≥ 33	197 (37.67)	3.44 ± 1.42	3.69 ± 1.19	48.67 ± 24.83	33.56 ± 14.60	20.09 ± 8.77	22.34 ± 9.30
**Smoking**							
Yes (ever)	260 (49.71)	3.32 ± 1.27	**3.81 ± 1.05^b^**	47.54 ± 24.89	34.60 ± 14.26	20.69 ± 8.64	23.03 ± 9.33
No (never)	263 (50.29)	3.34 ± 1.37	3.68 ± 1.24	47.64 ± 25.37	34.15 ± 14.19	20.39 ± 8.57	22.66 ± 9.16
**Drinking**							
Yes (ever)	207 (39.58)	**3.08 ± 1.10^c^**	3.81 ± 1.23	48.13 ± 25.31	34.52 ± 13.87	20.99 ± 8.53	23.29 ± 9.09
No (never)	316 (60.42)	3.49 ± 1.43	3.70 ± 1.16	47.24 ± 25.02	34.27 ± 14.46	20.24 ± 8.63	22.55 ± 9.34
**BMI**							
< 20	49 (9.37)	3.36 ± 1.25	3.93 ± 1.07	49.29 ± 25.72	34.49 ± 11.76	20.92 ± 7.39	23.31 ± 7.84
20–25	294 (56.21)	3.34 ± 1.34	3.68 ± 1.18	45.81 ± 24.09	34.38 ± 13.45	20.70 ± 8.25	22.89 ± 8.83
≥ 25	180 (34.42)	3.30 ± 1.31	3.80 ± 1.12	50.05 ± 26.45	**34.33 ± 16.06^d^**	**20.16 ± 9.47^d^**	**22.63 ± 10.26^d^**
**Abs**							
< 4	190 (36.33)	2.83 ± 0.97	3.63 ± 1.13	49.50 ± 25.74	34.15 ± 14.08	20.63 ± 8.76	22.85 ± 9.38
4–7	203 (38.81)	**3.49 ± 1.34**^e^	3.74 ± 1.08	48.26 ± 24.36	34.69 ± 13.66	20.96 ± 8.25	23.24 ± 8.95
≥ 7	130 (24.86)	**3.81 ± 1.49**^e^	**3.91 ± 1.28^e^**	**43.77 ± 25.12^e^**	34.20 ± 15.29	19.76 ± 8.87	22.24 ± 9.49

Values are mean ± SD unless otherwise stated.

VCL = curvilinear velocity; VSL = straight line velocity; VAP = average path velocity; BMI = body mass index; Abs = the duration of sexual abstinence.

^a^Values were re-transformed following Logarithmic transformation.

^b^*P* < 0.05 for comparison between the groups of smoking status.

^c^*P* < 0.05 for comparison between the groups of drinking status.

^d^*P* < 0.05 for comparison with the group of body mass index < 20.

^e^*P* < 0.05 for comparison with the group of abstinence time < 4 day.

Table 2 demonstrates the genotype frequency and sperm parameters of each variant. For the rs7004637, although the subjects with GG genotype showed lower motility, but when compared with the AA genotype, the difference did not reach the level of statistical significance (*P* = 0.092). For the rs16891278, the AG genotype carriers had significantly lower sperm concentration compared with the AA genotype (*P* = 0.044), but no statistical significance was found regarding those with the GG genotype, as well as the combination of AG and GG genotype. For the rs6773, the semen volume, concentration, motility, VCL, VSL and VAP were not significantly different between the genotypes of the variant.

**Table 2:** Sperm parameters according to the genetic variants of *VDAC3* gene in 523 males with definite idiopathic infertility

Variant	Genotype	*N* (%)	Semen volume	Concentration (106/ml)^a^	Motility (%)	VCL (μm/s)	VSL (μm/s)	VAP (μm/s)
rs7004637	AA	438 (83.75)	3.30 ± 1.27	3.75 ± 1.15	47.45 ± 25.42	34.30 ± 14.56	20.51 ± 8.82	22.82 ± 9.46
	AG	81 (15.49)	3.53 ± 1.55	3.75 ± 1.15	49.30 ± 23.17	35.22 ± 11.71	20.91 ± 6.98	23.27 ± 7.55
	GG	4 (0.76)	2.05 ± 0.19	2.91 ± 0.65	28.71 ± 26.39b	26.40 ± 20.66	16.58 ± 12.69	18.20 ± 13.88
	AG + GG	85 (16.25)	3.46 ± 1.55	3.72 ± 1.15	48.33 ± 23.57	34.77 ± 12.28	20.68 ± 7.31	23.01 ± 7.93
rs16891278	AA	389 (74.38)	3.34 ± 1.37	3.79 ± 1.09	46.67 ± 25.52	33.88 ± 14.61	20.13 ± 8.81	22.46 ± 9.48
	AG	119 (22.75)	3.31 ± 1.17	**3.52 ± 1.32^c^**	49.40 ± 24.45	36.11 ± 13.61	21.70 ± 8.22	23.96 ± 8.85
	GG	15 (2.87)	3.14 ± 1.13	4.29 ± 0.74	57.22 ± 16.36	33.32 ± 4.75	21.94 ± 3.89	23.95 ± 3.94
	AG+GG	134 (25.62)	3.29 ±1.17	3.61 ± 1.29	50.27 ± 23.76	35.80 ± 12.95	21.72 ± 7.85	23.96 ± 8.44
rs6773	CC	408 (78.01)	3.31 ± 1.27	3.76 ± 1.14	47.25 ± 25.51	33.95 ± 14.52	20.30 ± 8.77	22.60 ± 9.43
	CT	108 (20.65)	3.42 ± 1.49	3.73 ± 1.19	49.19 ± 23.47	36.29 ± 12.74	21.61 ± 7.79	23.99 ± 8.33
	TT	7 (1.34)	2.91 ± 1.10	3.23 ± 0.94	42.80 ± 28.23	30.66 ± 16.53	18.64 ± 9.47	20.44 ± 10.44
	CT + TT	115 (21.99)	3.39 ± 1.47	3.70 ± 1.17	48.80 ± 23.69	35.91 ± 13.00	21.41 ± 7.90	23.75 ± 8.47

All *P*-values were adjusted for age, smoking, drinking, body mass index (BMI) and the duration of sexual abstinence (Abs).

Values are mean ± SD unless otherwise stated.

VCL = curvilinear velocity; VSL = straight line velocity; VAP = average path velocity.

Statistical analysis was performed using multiple linear regression analysis.

^a^Values were re-transformed following Logarithmic transformation.

^b^*P* = 0.092 for comparison between rs7004637 GG and AA genotype.

^c^*P* < 0.05 for comparison between rs16891278 AG and GG genotype.

## DISCUSSION

This study represents the first attempt to investigate the impact of VDAC3 genetic variants on semen quality. Three tagSNPs (rs16891278, rs6773 and rs7004637) in the VDAC3 gene were selected using the tagSNP method. 523 Han-Chinese idiopathic infertility males were genotyped for these SNPs using TaqMan-based genotyping. Significant associations were detected between the genetic variant rs16891278 and sperm concentration. The other two SNPs do not affect semen quality. Since all the three SNP do not affect amino acid: rs16891278 (chr8:42401679–42401679) and rs7004637 (chr8:42392929–42392929) are both transcript variants occurring within the intron, while rs6773 locates at chr8:42405639–42405639 and is a UTR variant of the 3′ UTR. We suspect that rs16891278 may play a role at the transcription level. The characteristics of sperm number are one of the most informative parameters in semen quality analysis [27], and are dependent on normal spermatogenesis [28]. As the anion channel was involved in energy metabolism and cell apoptosis as well as distributed ubiquitously in germinal tissues and cells, it can be concluded that VDAC3 may play significant roles in the process of spermatogenesis.

This study found that the rs16891278 AG genotype carriers had significantly lower sperm concentration compared with the AA genotype. A possible explanation for this finding could be the participation of VDAC3 in the process of spermatogenesis. Outer mitochondrial membrane permeability is a central event in apoptotic cell death. VDAC interacted with Bcl-2 family members or with other proteins probably acts as a convergence point for a variety of life-or-death signals. When outer mitochondrial membrane permeability is increased, it releases several apoptogenic factors such as cytochrome c into the cytoplasm, which can activate the downstream destructive processes [12]. Previous study of Rahmani, et al. has showed that the hepatitis B virus produces a protein called X (HBV-X) that interacts with VDAC3 and induces apoptosis through forming a large pore with host VDAC3 plus some Bcl-2 family proteins such as Bax and Bak [29], thus VDAC3 can be involved in cell apoptosis through regulating mitochondrial membrane permeability. Therefore, too much sperm cell apoptosis may lead to sperm number reduced, and also may lead to lower sperm concentration. Additionally, as a physiological process, normal spermatogenesis needs energy supplied as well as a certain level of androgen maintained [30]. In germinal tissues and cells, VDAC3 protein expressed ubiquitously and particularly abundant in Leydig cell [14]. Evidences show that VDAC contains an ATP binding site and mediates ATP transport between extracellular environment and cytoplasm [31, 32]. It is plausible to believe that altered VDAC3 function caused by polymorphisms in the VDAC3 gene might affect energy supply for spermatogenesis and Leydig cell steroidgenesis, and at last affect spermatogenesis.

Although, in the study of bovine sperm, VDAC3 protein is observed to be localized abundantly in bovine outer dense fiber (ODF) of the flagella [15], which is hypothesized that the highly abundant VDAC proteins present in ODF might be involved in the maintenance and adaptation of ATP levels in the sperm flagellum, and thus involved in the regulation of sperm motility, there is no statistical association between sperm motility or its characteristics parameters (VCL, VSL and VAP) and variants in the VDAC3 gene in the study. Additionally, it is worthy of note that male mice lacking VDAC3 show markedly reduced sperm motility [14]. The inconformity to this study might be explained by that the moderately altered VDAC3 function by VDAC3 polymorphism could be sufficient only for decreasing sperm concentration through affecting spermatogenesis, but not for affecting the sperm motility or its characteristics parameters (VCL, VSL and VAP) like male mice lacking VDAC3. In the further study, we are researching for the relationship between VDAC3 and human sperm motility or semen quality from the perspective of epigenetics (e.g. DNA methylation). However, our study still has some limitations. As shown in Table 1, the sperm concentration of smoking group is higher than the non-smoking group. It's confusing to understand this result. Further studies with larger samples are necessary to elucidate the real impact of smoking on idiopathic male infertility. Also, the samples were obtained in andrology clinic. The health of wife was diagnosed by gynecologists. It's a pity that we did not collect the data of wife. In addition, the influence factors of infertility are very complex. It is hard to get true idiopathic male factor infertilities.

In conclusion, our present study showed that VDAC3 genetic variant, rs16891278, is associated with human sperm concentration. To our knowledge, it focuses on the relationship between VDAC genetic variants and human semen quality for the first time. Our findings would be beneficial to find more functions of VDAC in male reproductive system, to search for the cause and mechanism of some clinical idiopathic male infertility, and to explore new diagnostic and therapeutic methods for patients with poor semen quality.

## MATERIALS AND METHODS

### Subjects

From March 2014 to June 2015, a total of 523 ethnic Han-Chinese men visiting the Nanjing Maternity and Child Health Care Hospital Affiliated to Nanjing Medical University were consecutively recruited. They were all married and failed to conceive for at least 12 months. All patients were well-developed men with normal secondary sexual characteristics and testicular volume. The serum testosterone, LH, and FSH levels were within the normal range: testosterone = (3∼5.7) ng/mL, LH = (0.80∼5.10) mIU/mL and FSH = (0.80∼6.30) mIU/mL. Patients with orchitis, obstruction, cryptorchidism, varicocele, congenital bilateral absence of vas deferens, cytogenetic abnormalities, and Y chromosome microdeletions were excluded from the study after a complete historical and physical examination [33, 34]. They were diagnosed as idiopathic male infertility without an infertile wife.

Each patient provided informed consent and completed a questionnaire including information about age, smoking, drinking habits and other lifestyle factors. Besides, each subject donated 5 ml of peripheral blood for genomic DNA extraction and an ejaculate for semen analysis. This study protocol was approved by the ethics review board of Nanjing Medical University.

### Ethics statement

All participants gave written informed consent, and the study was approved by the Institutional Ethics Committee of Nanjing Medical University (Process n.2012/3).

### Semen analysis

According to World Health Organization guidelines (WHO, 1999), the computer-assisted semen analysis system (WLJY-9000; Weili New Century Science and Tech Dev., Beijing, China) was applied to perform the semen analysis. For the statistical analysis for an association with VDAC3 genetic variants, six parameters of semen quality were chosen: semen volume (ml) and sperm concentration (106/ml) (these two parameters represent sperm number); motility, curvilinear velocity (VCL, μm/s), straight line velocity (VSL, μm/s) and average path velocity (VAP, μm/s) (these four parameters indicate the sperm motility characteristics). The sperm numbers and sperm motility characteristics provided a reliable estimation of the fertilizing ability of human spermatozoa [35]. Strict quality control measures were enforced throughout the study. Values for semen parameters were the mean of at least two analyses.

### SNP selection

**Figure 1:**
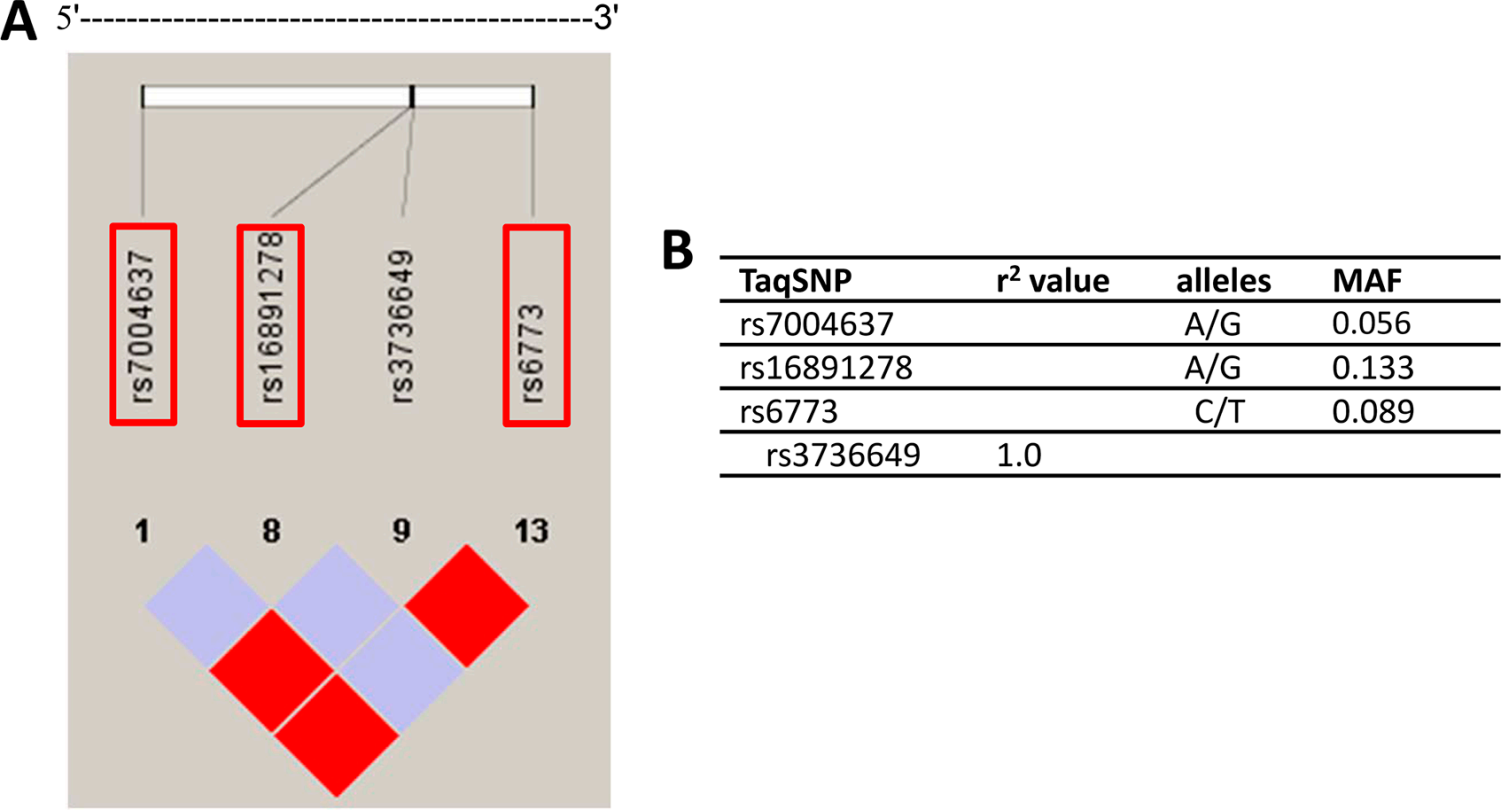
Overview of selected tagging single nucleotide polymorphisms (tagSNPs) and their characteristics (**A**) Location of the four SNPs within the *VDAC3* gene and 1500 base pairs (bp) upstream and 1500 bp downstream region. The selected tagSNPs are indicated by grey frame. (**B**) The four tagSNP and SNP that are indirectly measured by them are listed with corresponding *r*^2^ values. MAF = minor allele frequency.

Using genotype data obtained from unrelated Han Chinese in Beijing individuals in the HapMap (HapMap Data Rel 24/phase II nov08, on NCBI B36 assembly, dbSNP b126; http://hapmap.ncbi.nlm.nih.gov), the SNPs were selected within the 17023 bp human VDAC3 gene as well as 1500 bp upstream and 1500 bp downstream, which had a minor allele frequency > 0.05 in Han Chinese in Beijing and was pinpointed to chromosome 8 from 42367047 to 42384070, thirteen SNPs were captured in this region. A linkage disequilibrium (LD) plot of this region was made based on the r2 values, which indicate the ability of a certain SNP to predict another SNP [36], using Haploview 4.0 software. By applying Tagger and a tagging threshold of r2 > 0.80, three tagSNPs (rs7004637, rs16891278 and rs6773) were selected which represent other SNPs of this region with a mean r2 of 1.0 (Figure 1).

### Genotyping

Genomic DNA was extracted from peripheral blood leukocytes of 523 Han-Chinese men suffered infertility according to standard protocols (Genomic DNA kit; Tiangen, Beijing, China). TaqMan SNP Genotyping Assays were performed for genotyping using the Taq amplification method in a ABI Prism 7900 HT Fast Real-Time PCR system (Applied Biosystems, USA). PCR amplification was performed at 95°C for 10 minutes followed by 40 cycles at 95°C for 15 seconds, 56°C for 10 seconds and 60°C for 1 minute, with one additional cycle at 60°C for 10 minutes. Ten percent of study participants were randomly chosen and genotyped in duplicate to confirm the concordance of the genotyping results. In our study, all the call rates for these SNP genotyping were 100% and the concordance of duplicates was 100%.

### Statistical analysis

Computations were carried out using the Stata statistical package (Version 9.0, StataCorp, LP). Sperm concentration was undertaken logarithmic transformation to achieve homogeneity of variance and normal distribution of residuals. No transformation was performed for the remaining parameters. For statistical analysis, a multiple linear regression analysis was applied on the comparison of semen parameters that was considered for the genotypes of each SNP. Age, smoking, drinking, body mass index (BMI) and the duration of sexual abstinence (Abs) were initially considered as potential confounders. The *χ*2 test was used to evaluate differences in selected demographic variables, smoking and alcohol status. Continuous variables, including age and BMI, were evaluated by the analysis of variance (ANOVA). All results were reported as the mean ± SD, and *P* < 0.05 was considered to be significant.
